# Longevity-driven hepatic transcriptional programs mediate resilience to diet-induced liver injury in Ames dwarf mice

**DOI:** 10.1007/s11357-025-02017-6

**Published:** 2025-11-27

**Authors:** Jaspreet Kaur Osan, Sharlene Rakoczy, Heidi L. Pecoraro, Holly M. Brown-Borg

**Affiliations:** 1 Department of Biomedical Sciences, University of North Dakota, 504 Hamline St., Grand Forks, ND 58202 USA; 2 Veterinary Diagnostic Laboratory, North Dakota State University, Fargo, ND 58102 USA

**Keywords:** MASLD, Liver-transcriptome, High-fat diet, Long-living mice, Metabolism, GH signaling

## Abstract

The liver plays a central role in regulating systemic metabolism, and its function declines with age, contributing to increased susceptibility to metabolic diseases. Metabolic dysfunction-associated steatotic liver disease (MASLD), characterized by hepatic lipid accumulation and inflammation, is an early manifestation of liver dysfunction strongly associated with aging, insulin resistance, and high-fat diet (HFD) consumption. Ames Dwarf mice, which are growth hormone (GH)-deficient and long-lived, retain insulin sensitivity and exhibit resistance to age-related metabolic decline, making them an ideal model to study hepatic protection mechanisms. In this study, male and female Ames Dwarf and wildtype (WT) mice were fed either a standard diet or HFD for 12 weeks. WT males developed classical features of MASLD, including hepatic steatosis, hepatocyte ballooning, and elevated levels of inflammatory cytokines (IL-1β, MCP-1, IL-2, and IL-4). In contrast, Ames Dwarf mice exhibited minimal liver pathology, reduced lipid accumulation, and limited cytokine induction. Transcriptomic profiling revealed that WT mice upregulated genes involved in inflammation and proliferation, while Ames Dwarf mice showed activation of protective metabolic pathways (PPAR and AMPK) and suppression of lipogenic and fibrotic gene programs. Notably, female Ames Dwarf mice displayed the strongest resistance to HFD-induced changes, with minimal transcriptomic alterations. These findings suggest that disrupted GH signaling in Ames Dwarf mice leads to a reprogrammed hepatic response that preserves metabolic health and protects against MASLD, highlighting potential links between aging, GH signaling, and liver resilience.

**Supplementary Information:**

The online version contains supplementary material available at https://doi.org/10.1007/s11357-025-02017-6.

## Introduction

The liver plays a central role in maintaining metabolic homeostasis through processes such as gluconeogenesis, xenobiotic detoxification, and biosynthesis of key molecules [1]. However, with aging, hepatic function progressively declines, contributing to lipid accumulation—a hallmark feature of metabolic dysfunction-associated steatotic liver disease (MASLD) [2]. Recent projections indicate a dramatic rise in MASLD prevalence by 2050, with a predicted 300% increase among individuals aged 80 and older and a 58% increase among those aged 70–79 [3]. During this same period, the number of people over 80 is expected to rise from 6.5 million to 17.5 million, and those aged 65 and older from 58 to 82 million. This demographic shift will substantially elevate healthcare costs, primarily due to the growing burden of chronic, age-associated conditions like MASLD [4]. Understanding the biological mechanisms that drive MASLD—and identifying factors that may confer protection—is therefore critical to mitigating its future impact.

As the central hub of energy metabolism, the liver plays a direct role in glucose regulation through insulin signaling, making dietary intake a critical factor in shaping liver health over time [5]. One of the primary contributors to MASLD development is the consumption of a high-fat diet, a characteristic feature of the Western dietary pattern [6]. Such diets often lead to obesity, another major risk factor associated with MASLD [7]. Initially, excessive fat intake promotes de novo lipogenesis in adipose tissue, leading to increased fat storage. However, with continued high-fat intake, adipose tissue begins to undergo lipolysis, resulting in the release of free fatty acids into circulation. These free fatty acids are subsequently taken up by the liver, where they are converted into triglycerides, sterol esters, and ceramides—accumulating as lipid droplets within hepatocytes [8].

The excessive accumulation of these lipids, particularly ceramides and triglycerides, triggers liver injury by promoting immune cell infiltration, hepatocyte ballooning, and fibrosis [9]. These pathological changes mark the onset of liver damage, now classified as MASLD (formerly known as non-alcoholic fatty liver disease, or NAFLD) [10].

Sexual dimorphism is also associated with MASLD, with men more prone to MASLD as compared to women [11], and post-menopausal females tending to have increased susceptibility to MASLD [12]. In murine models, the cause of this sexual dimorphism has been investigated, with variation observed with respect to mouse breeds and duration of high-fat diet feeding [13–15]. On the contrary, in humans, it is linked to the level of sex hormones, where a decreased level of estrogen in women post-menopause leads to an increase in visceral fat and dyslipidemia [16]. Testosterone has a gender-specific impact, with increased serum testosterone in PCOS women leading to increased risk of MASLD and decreased testosterone in men with increased risk of MASLD [17].

Growth hormone (GH) is intertwined with the sex hormones via signaling pathways and with MASLD. GH deficiency is associated with the progression of MASLD, while administration of GH is linked to reduced steatosis and liver damage in patients [18]. MASLD is associated with obesity, where circulating GH is decreased and leads to an increased risk of MASLD. Growth hormone administration or inducing endogenous GH levels leads to increased IGF-1, which reduces steatosis and liver injury [19, 20]. The role of GH in the development of hepatic steatosis has been demonstrated using a liver-specific growth hormone receptor (GHR) knockdown mouse model. This model showed that loss of GH signaling directly affects STAT5b activity and indirectly influences IGF-1 levels, leading to increased de novo lipogenesis and reduced insulin sensitivity, both of which contribute to hepatic fat accumulation [21, 22].

Despite the documented benefits of growth hormone therapy in mitigating MASLD, accumulating evidence suggests that chronic GH deficiency may confer protection against high-fat diet–induced metabolic stress. A recent study using growth hormone–releasing hormone knockout (GHRH-KO) mice demonstrated that lifelong GH deficiency preserved insulin sensitivity and metabolic resilience, even when high-fat diets were introduced during mid-to-late life, while also extending lifespan [23]. Ames Dwarf mice are exceptionally long-lived mice [24], due to a mutation in the Prop-1 gene. The resulting growth hormone deficiency leads to insulin sensitivity and higher energy expenditure even when fed a high-fat diet [25]. GH plays a pivotal role in regulating insulin secretion, and in GH-deficient mice, insulin levels remain chronically low. This is significant because hyperinsulinemia, often seen in insulin-resistant states, is avoided, contributing to their preserved insulin sensitivity [26]. While GH therapy is known to reduce hepatic fibrosis through IGF-1–mediated suppression of lipogenesis [27], the paradox arises: why do GH-deficient models show protection, while GH supplementation appears therapeutic?

This discrepancy suggests that the biological effects of GH may differ depending on the context—short-term GH therapy versus lifelong GH deficiency. It raises the possibility that chronic GH deficiency establishes a unique metabolic and hormonal milieu that is inherently protective, distinguishing it from transient or therapeutic GH exposure. Understanding these differences may be key to optimizing interventions for MASLD. While previous studies have demonstrated the metabolic resilience of Ames Dwarf mice to high-fat diet (HFD) exposure, they did not investigate liver pathology—the defining clinical hallmark of MASLD. In this study, we examined liver pathology in Ames Dwarf mice after a 12-week high-fat diet regimen, assessing key histological features of MASLD. Additionally, we performed transcriptomic profiling of liver tissue to uncover gene expression changes associated with high-fat exposure. We hypothesize that Ames Dwarf mice will retain their unique metabolic phenotype under high-fat diet conditions, enabling the identification of key hepatic genes and pathways that contribute to either the development or protection from MASLD.

## Methods

### Animals and diets

Our lab at The University of North Dakota has maintained a colony of Ames Dwarf mice and their wild-type counterparts since 1996. The colony is housed under controlled conditions with a 12-h light/12-h dark photoperiod and a constant temperature of 22 ± 1 °C at the Center for Biomedical Research. Mice were housed in groups of 3–5 per cage. All procedures involving animals were reviewed and approved by the Institutional Animal Care and Use Committee (IACUC) at the University of North Dakota, in accordance with NIH guidelines for the care and use of laboratory animals.

Three- to four-month-old male and female Ames Dwarf and wildtype mice (N = 10–15 per group) were fed either a standard diet (Research Diets, Inc., catalog #D12450K) or a high-fat diet (60% kcal from fat; Research Diets, Inc., catalog #D12492) for 12 weeks.

### Tissue and plasma collection

At the end of the 12-week diet regimen, mice were euthanized using CO₂ inhalation. Body weights were recorded, and blood was collected via the intrahepatic vein. The collected blood was mixed with 0.5 M EDTA and centrifuged at 4000 RPM for 10 min at 4 °C. The supernatant (plasma) was collected and stored at −80 °C. Livers designated for histological analysis were perfused as described below, while livers for RNA sequencing were collected immediately after blood collection.

### Liver embedding and sectioning

The livers were perfused via the intrahepatic vein to eliminate blood from the organ. Subsequently, the livers underwent infiltration with 30% sucrose, followed by fixation in paraformaldehyde (PFA) and subsequent embedding in OCT media. Liver tissues (N = 5/group) were then cut into 10-µm thick sections using Leica CM3050S (Leica Biosystems; Deer Park, IL). Slides were stored at −80 °C for future analysis.

### Hematoxylin and eosin (H&E) staining

H&E staining was performed to assess immune cell infiltration and hepatocyte ballooning. Slides were brought to room temperature and loaded into the Autostainer XL (Leica ST5010) for automated staining, conducted by the UND Histology Core Facility.

### Picrosirius red staining

Picrosirius Red staining was performed to assess liver fibrosis and was conducted by the UND Histology Core Facility. A staining kit from STAT Lab (McKinney, TX) was used. Slides were brought to room temperature and fixed with zinc formalin for 30 min to prevent tissue detachment.

### Liver scoring

H&E and Picrosirius-stained slides were sent to North Dakota State University (NDSU) pathology department for pathology scoring. Pathology scoring was done based on the NASH clinical research network scoring system [28].

### Oil red O staining

Oil Red O staining was performed to visualize intracellular lipid droplets. Slides were fixed with Bouin’s fluid for 2–5 min, followed by staining using the Oil Red O Stain Kit from StatLab (Cat# KTORO). Images were acquired using an automated slide imager. For each slide, five random fields were captured at 20X magnification, and lipid droplet sizes were quantified using ImageJ [29].

### Cytokine multiplex assay

Plasma was used to measure circulating cytokines via the Rayplex Mouse inflammation bead array (Cat# FAM-INF-1) and Raybiotech Quantibody Mouse Cytokine Array 1 (Cat# QAM-CYT-1). Assays were performed according to the manufacturer's guidelines. The values above and below detection limits were not plotted.

### RNA-seq analysis

RNA was extracted from liver tissue (n = 3 per genotype, sex, and diet) using the Qiagen AllPrep DNA/RNA/Protein Mini Kit (Cat# 80,004). RNA integrity was assessed using an Agilent TapeStation, and only samples with RIN values greater than 7 were selected for library preparation. RNA sequencing was performed at Diagenode for the female samples (Denville, NJ) and at the UND Genomic Core Facility for the male samples. The sequencing was conducted on an Illumina NovaSeq 6000 instrument, generating 50 bp paired-end reads with Control Software 1.7.0. The sequencing data quality was assessed using FastQC, and subsequent trimming was performed using Cutadapt v3.5. The processed sequences were aligned using the STAR aligner version 2.7.9a. RNA abundance was estimated using MGCount, and data variability was visually represented through principal component analysis. Differential expression analysis was performed using DESeq2 to identify significantly regulated genes between conditions. Raw RNA-Seq counts were normalized, and genes with low expression were filtered out. Differentially expressed genes (DEGs) were identified based on an adjusted p-value (padj < 0.001) and an absolute log2 fold change > 1.5. Volcano plots were plotted using RStudio. Gene Ontology (GO) enrichment analysis, KEGG pathway analysis were performed using Shinygo [30].

### Statistics

The graphs were plotted using GraphPad Prism. Two-way ANOVA with Sidak multiple comparison tests were performed using GraphPad Prism version 10.2.3 for Windows (GraphPad Software, www.graphpad.com). P values < 0.05 were considered significant.

## Results

### Ames dwarf mice showed minimal liver damage irrespective of sex

To assess liver damage induced by a high-fat diet, both Ames Dwarf and wildtype (WT) mice were fed either a standard diet or HFD for 12 weeks. While all mice on the HFD showed a significant increase in body weight (Supplementary Fig.  1A), only male WT mice exhibited an elevated liver-to-body weight ratio (Supplementary Fig. 1B).

**Fig. 1:**
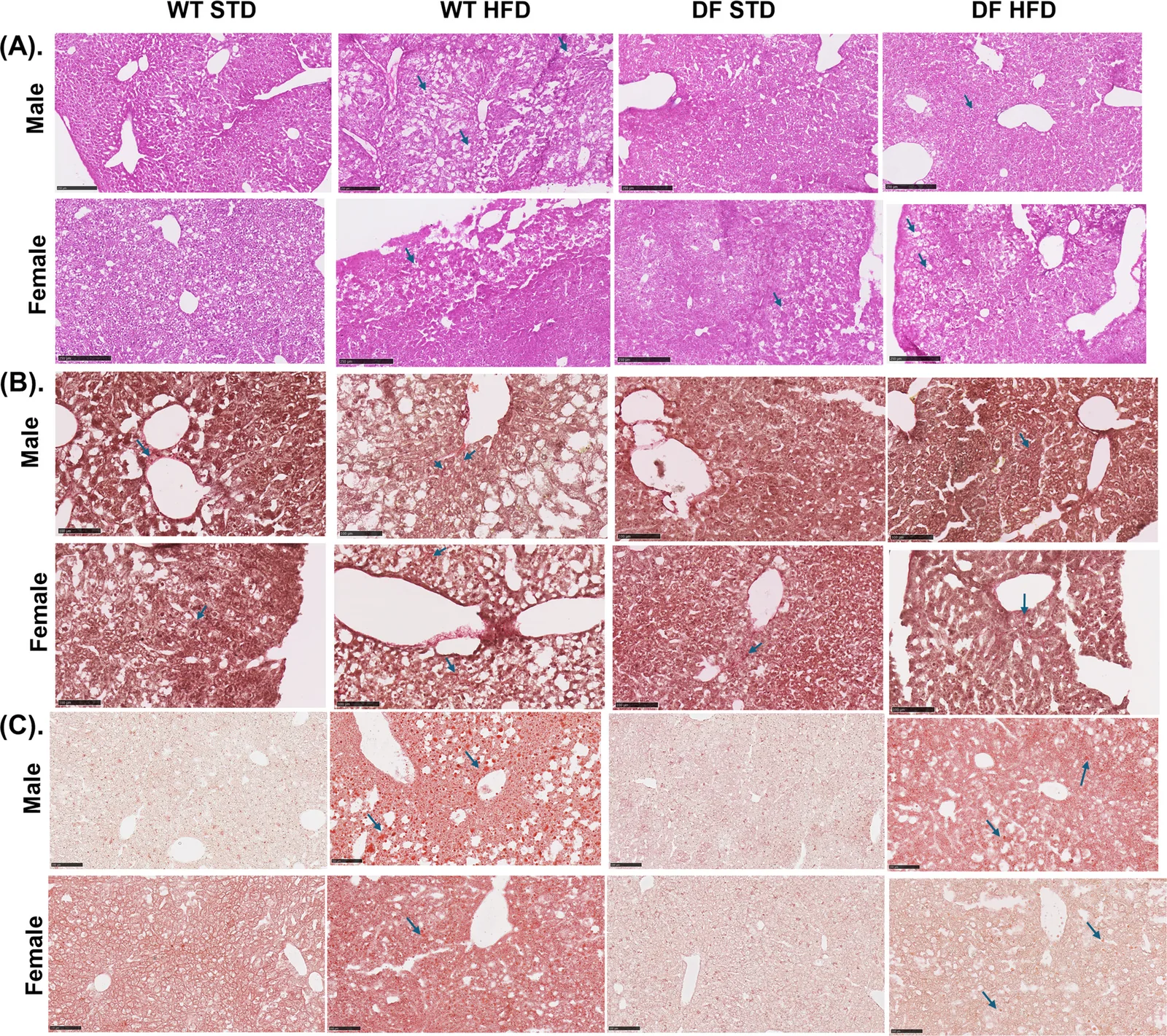
Histological analysis of liver tissue of male and female wild-type mice and Ames Dwarf mice fed on a standard diet or high-fat diet for 12 weeks. (**A**). H & E staining showing hepatocyte ballooning (**B**). Sirius red staining showing fibrosis (**C**). Oil-red-o staining showing lipid droplet deposition

**Fig. 2:**
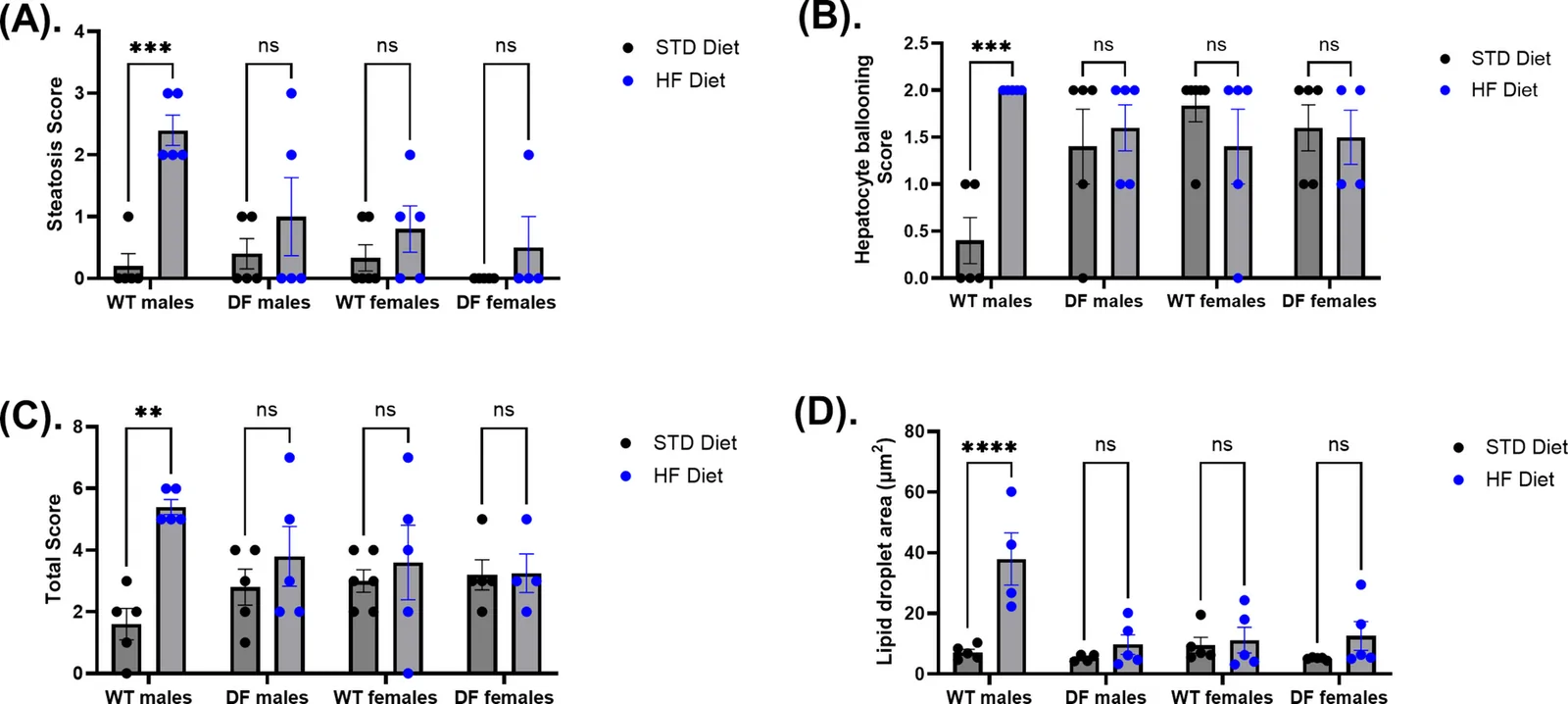
Histological analysis of liver derived from male and female Wildtype and Ames Dwarf mice fed a standard or high-fat diet for 12 weeks. Graphs for (**A**). Steatosis score (**B**). Hepatocyte ballooning score (**C**). Total pathology score (**D**). The lipid droplet area is plotted, and data (n = 5) is segregated based on sex and diet. Data is analyzed using two-way ANOVA. *p < 0.05; **p < 0.01; ***p < 0.005; ****p < 0.001

Histopathological analysis was performed using H&E and Sirius Red staining. Liver damage was scored based on hepatocyte ballooning (Fig. 1A), fibrosis (Fig. 1B), steatosis, and lobular inflammation. A total liver damage score was calculated by summing individual scores. Male WT mice on a HFD exhibited significantly increased steatosis (Fig. 2A), hepatocyte ballooning (Fig. 2B), and total liver damage score (Fig. 2C) compared to standard diet controls. No significant differences were observed in fibrosis (Supplementary Fig.  1C) or lobular inflammation (Supplementary Fig. 1D). Female WT mice on a HFD did not show significant liver damage relative to controls. Notably, Ames Dwarf mice, regardless of sex, exhibited no significant liver pathology in response to the HFD.

To further assess lipid accumulation, liver sections were stained with Oil Red O, and lipid droplet size was quantified using ImageJ software [29]. Male WT mice on high fat showed a significant increase in the size of lipid droplet deposition (Figs. 1C, 2D) compared to standard diet-fed controls. In contrast, female WT mice did not show significant changes in lipid accumulation. Consistent with histological findings, Ames Dwarf mice of both sexes exhibited no significant lipid accumulation in response to the HFD.

### MCP-1 and IL-1b are key inflammation drivers in male WT mice

**Fig. 3:**
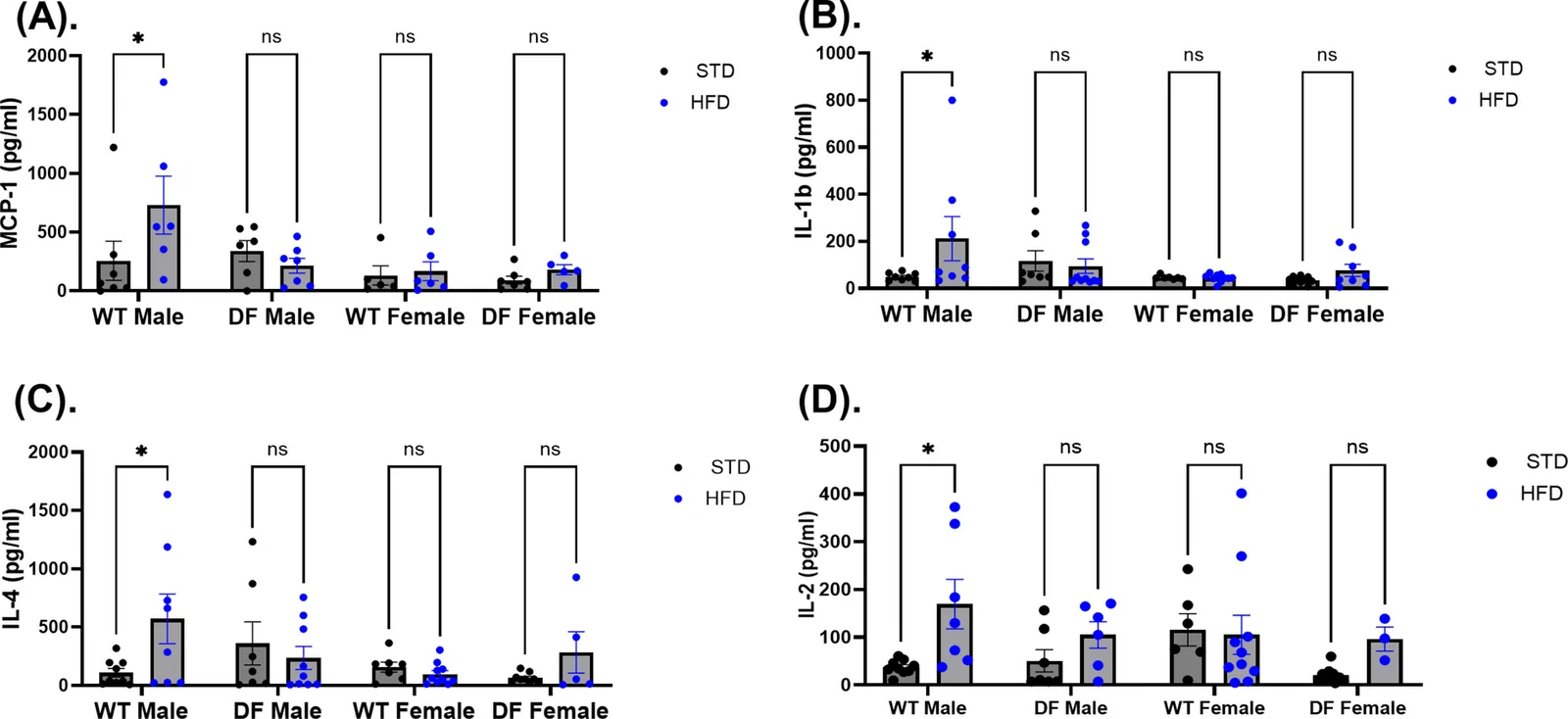
Inflammatory cytokines were measured in the plasma of the wildtype and Ames Dwarf mice fed a standard and high-fat diet for 12 weeks by using Raybiotech multiplex assay. Data (n = 7) were plotted for (A). IL-1b (B). MCP-1(C). IL-4 (D). IL-2 and data are segregated based on the sex of the mice and diet. Data is analyzed using two-way ANOVA. *p < 0.05; **p < 0.01; ***p < 0.005; ****p < 0.001

To investigate systemic inflammation, plasma samples collected after 12 weeks of HFD feeding were analyzed using a multiplex cytokine assay profiling 20 inflammatory markers. Among these, MCP-1 and IL-1β—well-established inflammatory mediators associated with MASLD [31, 32]—were significantly elevated in male WT mice on an HFD (Fig. 3A, 3B) compared to their standard diet counterparts. Additionally, increased levels of IL-4 and IL-2 (Fig 3C, 3D) were observed in the same group. In contrast, female WT mice and Ames Dwarf mice of both sexes exhibited no significant changes in plasma cytokine levels between dietary groups, indicating a sex- and genotype-specific inflammatory response to HFD exposure.

### Liver transcriptome reveals distinct cellular and metabolic responses in Ames dwarf mice

To investigate baseline transcriptomic differences, bulk RNA sequencing was performed on liver samples from Ames Dwarf and WT mice maintained on a standard diet. Differential gene expression analysis using DESeq2 was conducted with a significance threshold of Padj < 0.001 and |log₂ fold change|> 1.5 (Supplemental data).

**Fig. 4:**
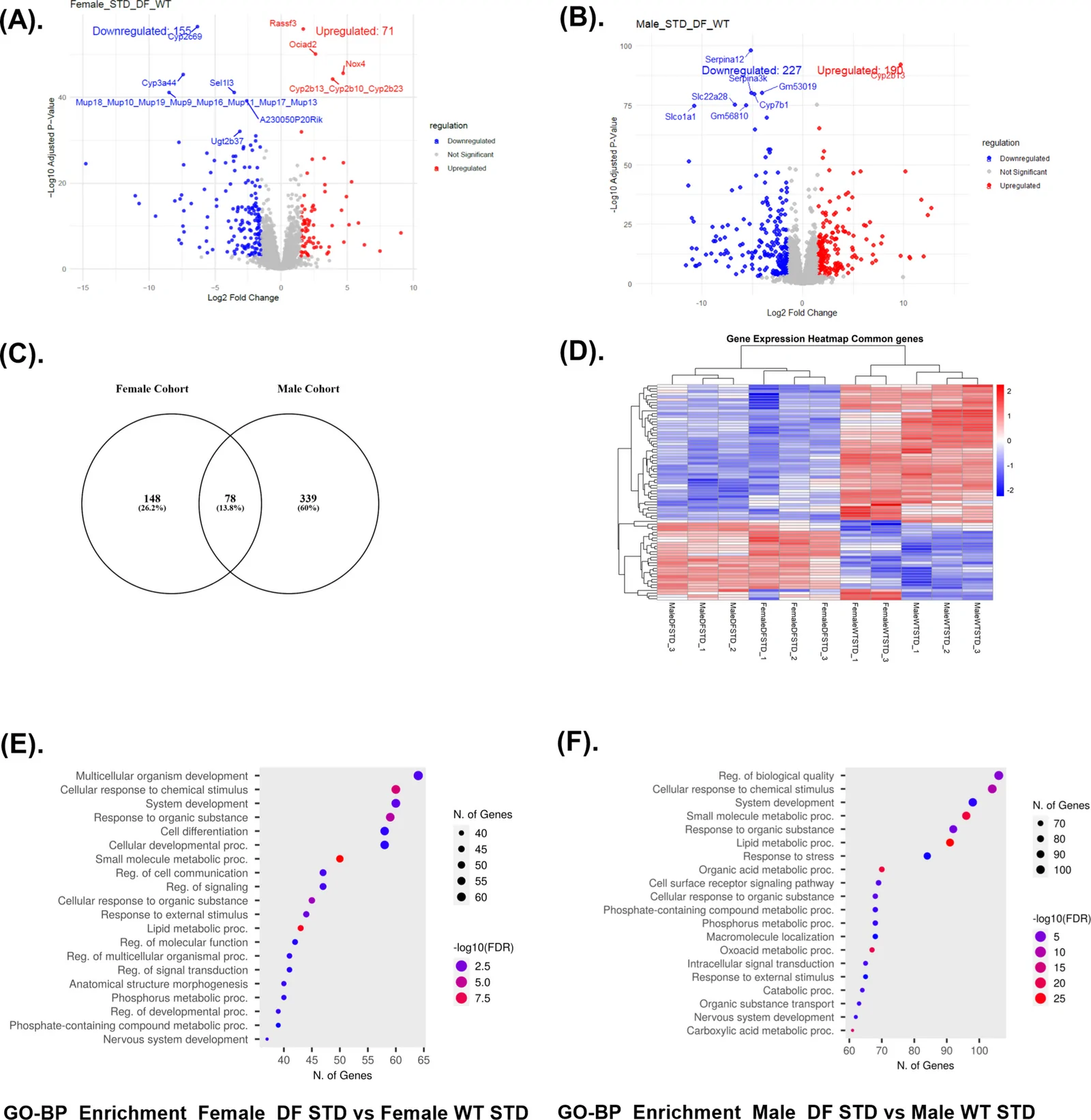
Comparison of whole genome sequencing of liver tissue from wildtype and Ames Dwarf mice on the standard for 12 weeks. The Volcano plot is plotted, genes with log twofold with a minimum change of 1.5, and Padj < 0.001 plotted. (**A**). Female mice, Dwarf vs Wildtype on a standard diet (**B**). Male mice, Dwarf vs Wildtype on a standard diet (**C**). Venn diagram showing the common and exclusive number of differentially expressed genes between the female (Fig. 4a) and male (Fig. 4b) (**D**). Heatmap showing common gene expression across all the samples by plotting log 2 of counts. Gene ontology Biological process pathway enrichment using ShinyGo analysis is shown for (**E**). Genes exclusively expressed in the female group (F). Genes exclusively expressed in the male group

Compared to WT controls, female Ames Dwarf mice exhibited 155 downregulated and 71 upregulated genes (Fig. 4A), while male Ames Dwarf mice showed 227 downregulated and 190 upregulated genes (Fig. 4B). The top differentially expressed genes in both sexes were primarily associated with lipid metabolic pathways. In female Ames Dwarf mice, prominent genes included members of the cytochrome P450 family—*Cyp2b10, Cyp2b13, Cyp2b23*, and *Cyp3a44*—which are involved in arachidonic acid and linoleic acid metabolism. In male Ames Dwarf mice, genes such as *Elovl3* and *Cyp2b13*, linked to unsaturated fatty acid metabolism, were notably enriched.

A Venn diagram comparing differentially expressed genes in both sexes revealed 78 genes (13.8%) shared between groups, 148 genes (26.2%) unique to females, and 339 genes (60%) unique to males (Fig. 4C). Heatmap analysis of the shared genes revealed consistent expression patterns between Ames Dwarf and WT mice, regardless of sex (Fig. 4D).

To explore functional implications, gene ontology-biological process (GO-BP) enrichment analysis was conducted using ShinyGO on sex-specific gene sets. The most enriched pathways (FDR < 0.05), sorted by gene count in females (Fig. 4E) and males (Fig. 4F), included cellular response to chemical stimuli, system development, response to organic substances, small molecule metabolic processes, and lipid metabolism.

Despite sex-specific differences in the number of differentially expressed genes, both male and female Ames Dwarf mice exhibited enrichment in similar biological processes, suggesting conserved cellular and metabolic adaptations that may contribute to their unique physiology.

### Female mice exhibit minimal transcriptomic changes in response to a high fat diet

**Fig. 5:**
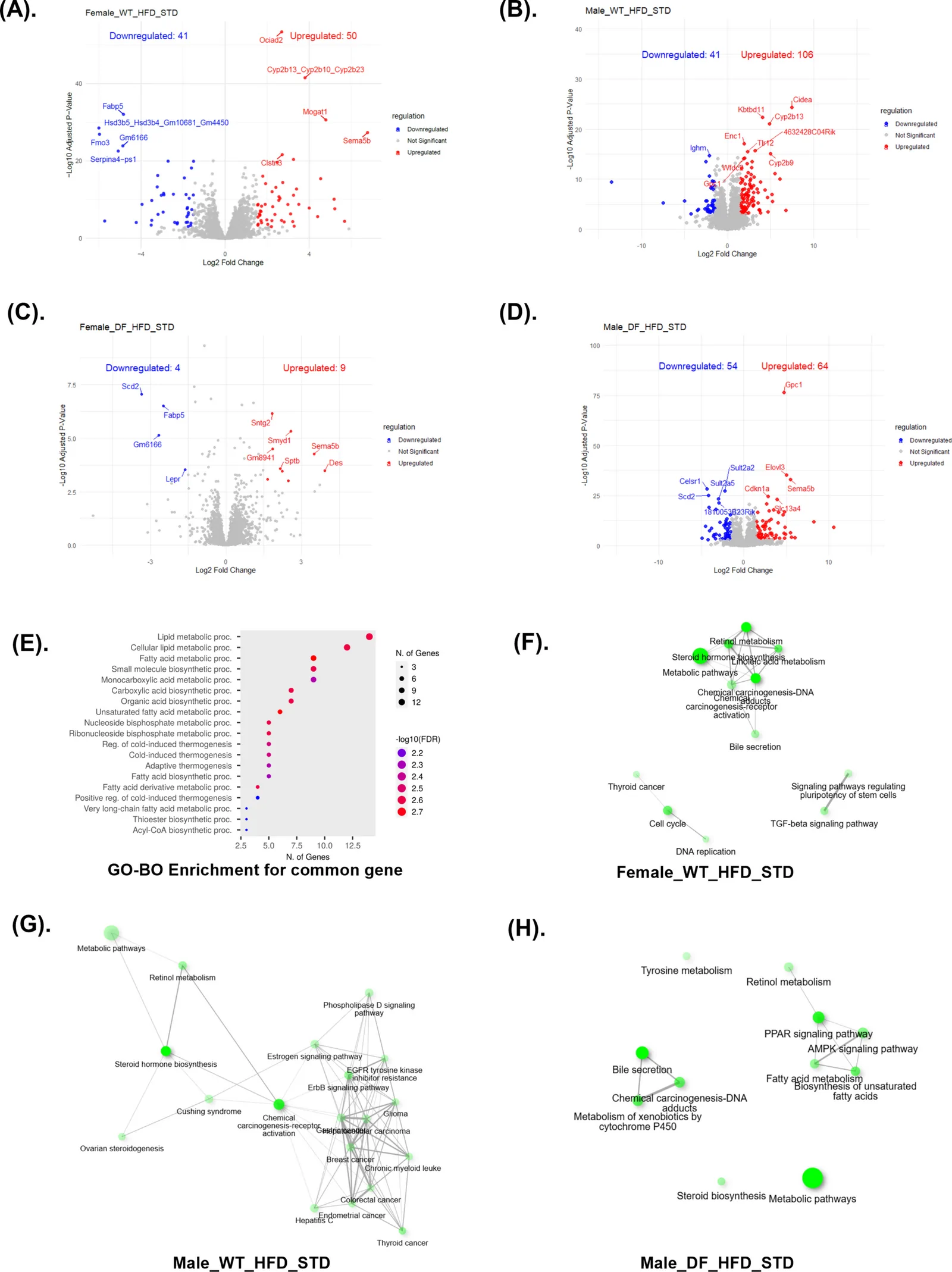
Comparison of whole genome sequencing of liver tissue from wildtype and Ames Dwarf mice on the standard and high-fat diet for 12 weeks. Groups were classified by comparing the high-fat diet to the standard diet. Volcano plot is plotted and genes with log twofold with a minimum change of 1.5 and Padj < 0.001. (**A**). Female wildtype high-fat diet vs standard diet (**B**). Male wildtype high-fat diet vs standard diet (**C**). Female dwarf high-fat diet vs standard diet (**D**). Male dwarf high-fat diet vs standard diet. (**E**). Gene ontological Biological process pathway enrichment for common genes (**F**). Network analysis of KEGG pathways for genes exclusively present in female WT HFD vs WT STD (**G**). Network analysis of KEGG pathways for genes exclusively present in male WT HFD vs WT STD (**H**). Network analysis of KEGG pathways for genes exclusively present in male DF HFD vs DF STD

To assess the impact of a HFD on liver gene expression, bulk RNA sequencing was performed on liver samples from both WT and Ames Dwarf mice of both sexes, maintained on either a standard or a HFD for 12 weeks. Female mice, regardless of genotype, exhibited minimal transcriptomic alterations in response to the HFD. Specifically, female WT mice displayed 50 upregulated and 41 downregulated genes compared to their standard diet counterparts (Fig. 5A), while female Ames Dwarf mice exhibited only 9 upregulated and 4 downregulated genes (Fig. 5C).

In contrast, male mice showed a more pronounced response. Male WT mice on a HFD had 106 upregulated and 41 downregulated genes (Fig. 5B), approximately 1.5 times more than their female counterparts. Male Ames Dwarf mice exhibited 64 upregulated and 54 downregulated genes (Fig. 5D), more than tenfold the number of differentially expressed genes seen in female Ames Dwarf mice.

These results indicate that female mice, particularly Ames Dwarf females, exhibit a blunted hepatic transcriptomic response to HFD, suggesting a potential sex-dependent protection mechanism against HFD-induced metabolic stress in the liver.

### Common gene analysis reveals shared hepatic response to HFD in female WT and male Ames dwarf mice

To investigate shared hepatic transcriptional responses to HFD, we compared differentially expressed genes between female WT, male WT, and male Ames Dwarf mice on a HFD relative to their respective standard diet controls. Six genes were found to be commonly differentially expressed across all three groups (Supplementary Fig.  2 A), including key genes related to lipid metabolism, such as *Clstn3*, *Gpc1*, and *Plin4*. Additionally, 16 genes were shared between male and female WT mice, 10 genes were shared between female WT and male Ames Dwarf mice, and 28 genes were shared between male WT and male Ames Dwarf mice.

A heatmap of log2-transformed expression values for these shared genes (Supplementary Fig. 2B) revealed distinct clustering patterns. WT males and females on a standard diet were grouped, whereas female WT and male Ames Dwarf mice fed a HFD clustered more closely, suggesting a convergent transcriptional response to HFD-induced metabolic stress between these two groups.

Gene ontology enrichment analysis of the shared genes revealed the top 20 biological processes, including several pathways related to lipid metabolism and small molecule processing (Fig. 5E), based on fold enrichment and FDR values. These findings indicate that although the expression levels of the shared genes did not differ drastically across groups, subtle shifts in gene expression under HFD conditions led to a shared molecular signature between female and male Ames Dwarf mice. This clustering may reflect similar protective or adaptive mechanisms to high-fat diet exposure in these groups.

### Pathway enrichment reveals PPAR-AMPK axis modulation in male Ames dwarf mice fed high fat

To further understand the transcriptional adaptations to HFD exposure, KEGG pathway enrichment analysis was performed on uniquely expressed genes in each group. In female wildtype mice fed a HFD, 59 unique genes were identified, which mapped to the top 12 significantly enriched KEGG pathways (Fig. 5F). These pathways formed three distinct functional clusters: metabolic processes, cell cycle regulation, and physiological homeostasis.

In male wildtype mice, 97 unique genes were used for enrichment analysis. The resulting KEGG pathways clustered into a single group, primarily enriched for cancer-related pathways, including those related to hepatocellular carcinoma (Fig. 5G). Notably, key genes such as *Gadd45a*, *Egfr*, and *Myc*, were upregulated. *Myc* is known to be associated with inflammation and cell cycle dysregulation [33], suggesting that male wildtype mice exhibit a transcriptional response indicative of cellular stress and potential oncogenic signaling under HFD conditions.

In contrast, high-fat-fed male Ames Dwarf mice exhibited a unique transcriptional profile, characterized by 74 distinct genes. KEGG pathway analysis revealed two prominent functional clusters, with the most enriched cluster involving peroxisome proliferator-activated receptor (PPAR) signaling and AMPK signaling pathways (Fig. 5H). Critical metabolic regulators, including *Scd1*, *Scd2*, *Acaca*, Acacb, *Irs2*, and *Ugt1a5* were downregulated in Ames Dwarf mice, suggesting that modulation of the PPAR-AMPK axis contributes to their resilience against HFD-induced metabolic stress. These findings support the hypothesis that Ames Dwarf mice are protected from HFD-mediated liver damage through coordinated downregulation of lipid biosynthesis and enhanced metabolic regulation [34].

### Ames dwarf mice on a HFD exhibit altered lipid metabolic processes compared to wildtype mice

**Fig. 6:**
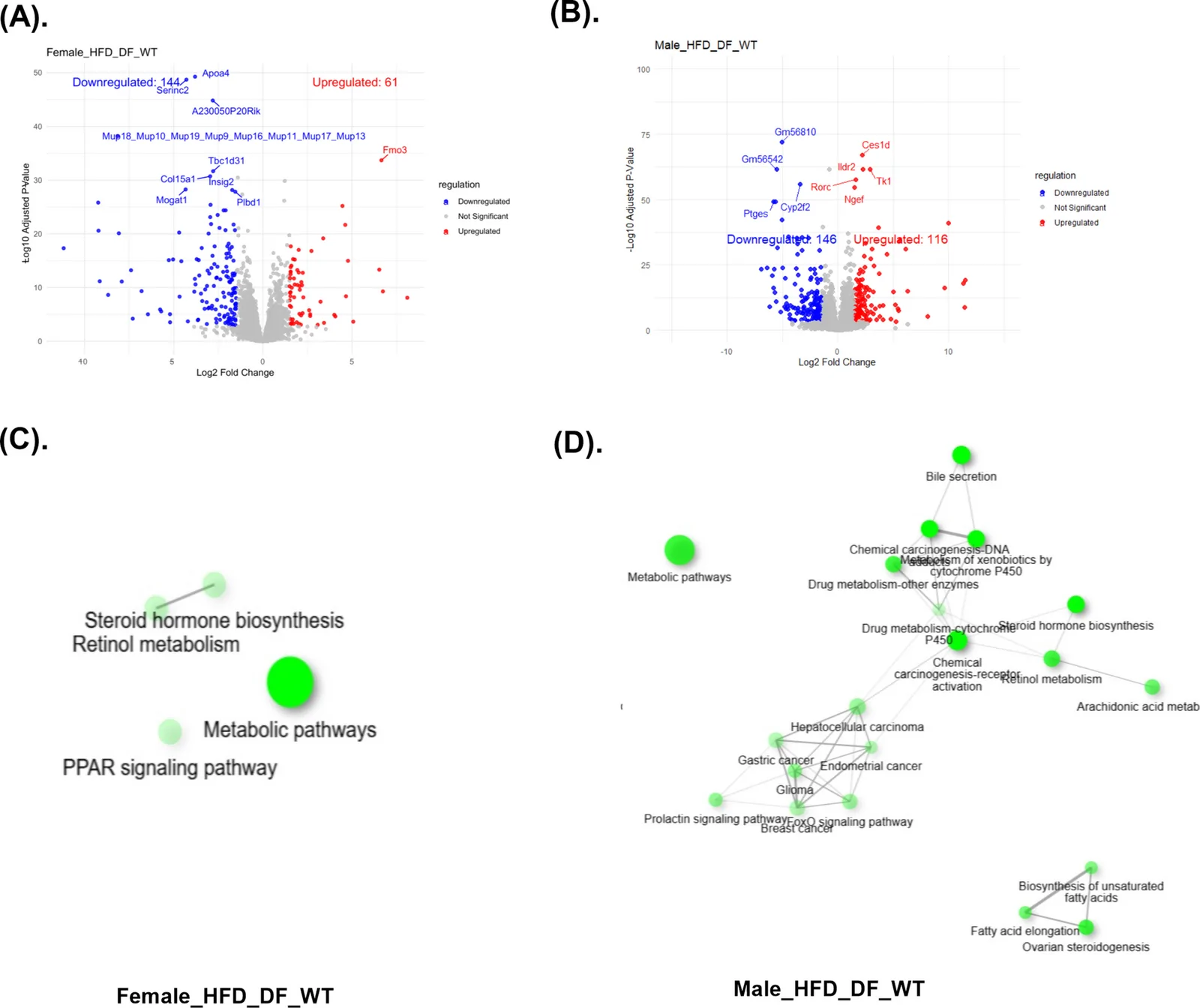
Comparison of whole genome sequencing of liver tissue from wildtype and Ames Dwarf mice on the high-fat diet for 12 weeks. A volcano plot is generated for female or male Ames Dwarf mice compared to wildtype mice on the high-fat diet. (**A**). High-fat diet female Ames Dwarf mice vs. female wildtype mice (**B**). High-fat diet male Ames Dwarf mice vs. male wildtype mice. (**C**). Network analysis of KEGG pathways for genes exclusively present in female DF HFD vs WT HFD (**D**). Network analysis of KEGG pathways for genes exclusively present in male DF HFD vs WT HFD

To determine how Ames Dwarf mice differ from wildtype mice in their response to high fat, we performed transcriptomic comparisons between these genotypes. Female Ames Dwarf mice fed high fat, relative to female wildtype mice, exhibited 144 downregulated and 61 upregulated genes (Fig. 6A). In male Ames Dwarf mice, 116 genes were downregulated and 146 upregulated compared to their wildtype counterparts on the same diet (Fig. 6B).

A comparison of the differentially expressed genes between male and female Ames Dwarf mice revealed 52 common genes (12.5%) across both sexes (Supplementary Fig.  2 C), with 153 genes uniquely regulated in females and 210 in males. Heatmap analysis based on log₂ expression values of these common genes (Supplementary Fig. 2D) revealed distinct clustering of Ames Dwarf and wildtype mice, suggesting consistent transcriptomic divergence between genotypes under HFD conditions. Among the commonly upregulated genes, *Lamb3*, *Adgrv1*, and *Elovl3* were notably elevated in male Ames Dwarf mice as well as in wildtype mice of both sexes.

To explore the functional relevance of these changes, we performed KEGG pathway enrichment using ShinyGO. In female Ames Dwarf mice, 153 unique genes enriched pathways that grouped into three distinct clusters: metabolic pathways, PPAR signaling, and retinol metabolism linked to steroid hormone biosynthesis (Fig. 6C). In male Ames Dwarf mice, 210 unique genes were identified in enriched pathways associated with metabolic processes, fatty acid metabolism, and cancer-related pathways, including those connected to drug metabolism (Fig. 6D). These findings indicate sex-specific transcriptional responses in Ames Dwarf mice, with both groups showing a strong enrichment of lipid metabolism and PPAR-related pathways, consistent with their resistance to HFD-induced liver damage.

### Transcriptome profiles suggest the balance between inflammation and metabolism determines hepatotoxicity

Pathway enrichment analysis revealed that male WT, female WT, and male Ames Dwarf mice fed high-fat diets shared several enriched biological pathways when compared to their STD counterparts. However, histological evaluation demonstrated reduced hepatic damage in male and female Ames Dwarf mice, indicating that pathway enrichment alone does not fully explain the phenotypic outcomes. To further understand this discrepancy, we examined individual gene expression within these shared pathways and assessed their association with MASLD based on existing literature.

In male WT mice on a HFD, several genes—*Uap1l1*, *Pde4d*, *Mthfd1l*, *Smpd3*, and *Myc*—were expressed in a direction consistent with enhanced inflammatory signaling and liver fibrosis (Table 1), as previously reported [33, 35–38]. Several genes involved in lipid signaling and metabolic pathways, including *PPARγ, Acot3, Abcc3, Smpd3,* and *Cidec*, showed expression patterns that correlated with NAFLD patient scores [38–41]. The expression direction of these genes is consistent with previous literature, indicating their roles in exacerbating disease progression (Table 1). Conversely, in the female cohort, genes such as *Vnn1*, *Etnppl*, and *Mogat1* were upregulated (Table 1). These genes are implicated in the dysregulation of gluconeogenesis and lipid metabolism, but are not strongly associated with fibrosis, potentially explaining the observed weight gain in females without accompanying hepatic damage.

Importantly, in male DF mice fed a high-fat diet, most of the differentially expressed metabolic genes—including *Acaca, Acacb, Cyp4a12a, Cyp4a12b, Fdps, Scd1,* and *Scd2*—were regulated in a manner consistent with protection against liver fibrosis [34, 42–45]. (Table 1). This suggests a coordinated transcriptional response that favors metabolic homeostasis and limits inflammatory damage, ultimately contributing to the hepatoprotective phenotype observed in Ames Dwarf mice.

## Discussion

Growth hormone (GH) signaling is a key regulator of metabolism, and its deficiency—observed in Ames Dwarf mice—has been associated with extended lifespan and metabolic protection [24, 46]. MASLD, a condition characterized by hepatic lipid accumulation and inflammation, often worsens with age and is influenced by GH activity through STAT5b-mediated regulation of de novo lipogenesis [22]. While GH supplementation reduces hepatic steatosis, paradoxically, GH-deficient models like GHRH-KO and Ames Dwarf mice exhibit resilience to diet-induced steatosis [22, 23, 25, 47]. However, comprehensive histological evaluation of liver injury in these models has been limited. Our study bridges this gap by integrating histopathology, cytokine profiling, and transcriptomics to evaluate the hepatic response to HFD in Ames Dwarf mice.

Consistent with prior metabolic phenotyping, we found that Ames Dwarf mice, regardless of sex, were protected from classical histological features of MASLD. WT males on HFD showed clear signs of steatosis and hepatocyte ballooning—hallmarks of liver injury [48]. In contrast, Dwarf mice exhibited minimal or no histological damage, and Oil Red O staining confirmed reduced lipid deposition. These results suggest a fundamental protective mechanism that mitigates the hepatotoxic effects of lipid overload in Ames Dwarf mice.

Hepatic inflammation is a central factor in the progression of MASLD. Ballooning degeneration of hepatocytes is often linked to oxidative stress and pro-inflammatory cytokines like IL-1β, MCP-1, and TNF-α [31, 32, 49, 50]. In our study, male WT mice on HFD showed increased IL-1β and MCP-1 expression, indicative of heightened inflammatory stress, while TNF-α levels were not significant (data not shown), but did show a trend of increase on HFD in WT mice. IL-1β upregulates fatty acid synthase, which promotes hepatic lipogenesis and contributes to liver steatosis formation due to excessive deposition of triglycerides and lipid droplets in the liver [31]. It also upregulates ICAM expression via liver sinusoidal endothelial cells, which promotes inflammation [51]. We also observed an increase in IL-2 and IL-4 levels in male WT mice fed HFD. Although the roles of IL-2 and IL-4 in MASLD are not well characterized, both cytokines are known to play a role in immune regulation. IL-2 primarily promotes the proliferation and activation of T cells and other immune effector populations, while IL-4 plays a key role in modulating natural killer T (NKT) cell function and shaping Th2-type immune responses. Their upregulation may reflect a compensatory immune mechanism or a shift in the hepatic immune microenvironment in response to HFD-induced metabolic stress [52]. Importantly, Ames Dwarf mice did not exhibit significant elevations in any of these cytokines, further supporting their immunometabolic resilience.

In male WT mice fed a high fat diet, KEGG pathway analysis revealed an enrichment of cancer-related signaling pathways. The genes contributing to this pathway included *Egfr, Gadd45a, Pparγ, Myc, and Nrg1*. Among these, *Myc* was notably upregulated in the WT-HFD group, consistent with its role in promoting liver regeneration and suggesting a compensatory response to HFD-induced hepatic injury. In contrast, *Egfr* expression was downregulated, which may act counter to Myc by reducing hepatocyte proliferation. Additionally, *Nrg1* and *Gadd45a* appeared to exert compensatory effects by inhibiting IL-6 and TGFβ signaling, respectively. Together, these findings suggest that exposure to a high-fat diet induces both injurious and compensatory molecular responses within the liver.

*PPARγ* was also upregulated, and although it is often associated with tumor suppression by maintaining a balance between pro- and anti-inflammatory cytokines, in the context of metabolism, its upregulation under high-fat diet conditions promotes hepatic steatosis [39, 53]. The progression of steatosis was further supported by the increased expression of *CD36*, a fatty acid uptake transporter whose expression is positively regulated by *PPARγ*. Additionally, *Pde4d* expression was elevated, and its upregulation has been linked to enhanced *CD36* expression and increased lipid accumulation in the liver [36], collectively contributing to the development of hepatic steatosis.

Next, there were genes involved in metabolic pathways that were also associated with promoting inflammation. This includes *CYP1A2*, which is downregulated during sepsis and shown to be directly associated with increased pro-inflammatory cytokines, including IL-1β, TNF, and IL-6 [54]. *Aldh3a2* was upregulated and is associated with increased triglyceride accumulation due to its role in converting long-chain aliphatic aldehydes into fatty acids [55, 56]. Another gene of interest was *Hsd3b5*, a male-specific gene involved in testosterone biosynthesis. *Hsd3b5* can indirectly influence lipid metabolism through modulation of the PPAR signaling pathway. Given that elevated testosterone levels are a known risk factor for hepatocellular carcinoma (HCC), dysregulation of *Hsd3b5* may contribute to sex-specific susceptibility to liver disease progression [57, 58]. Taken together, the pathological findings, cytokine profiles, and transcriptomic data collectively indicate the presence of liver injury in male WT mice on HFD. WT female mice exhibited significant transcriptomic alterations in response to HFD yet lacked the overt histological liver damage observed in males. This dissociation between gene expression and tissue pathology may be attributed, in part, to estrogen signaling, which is known to modulate genes involved in lipid metabolism, cholesterol transport, and mitochondrial function—mechanisms that confer protection against hepatic steatosis [12, 16]. Furthermore, the duration of HFD exposure may influence the onset of pathology, as prolonged exposure could potentially overcome estrogen-mediated protection [14]. This may explain why transcriptomic, but not histological, changes were detected within the timeframe of our study.

In contrast, female Ames Dwarf mice displayed the most profound resistance to HFD-induced hepatic alterations. These mice exhibited no significant histological damage, lipid accumulation, elevation of inflammatory cytokines, or substantial transcriptomic shifts. Only 13 genes were differentially expressed in female Ames Dwarf mice fed high fat compared to those on a standard diet. Among them, *Scd2*, *Vnn1*, *Lepr*, and *Fabp5* were particularly notable for their roles in lipid metabolism and immune regulation. Interestingly, these genes exhibited similar expression profiles in WT females and male Ames Dwarf mice on HFD, but not in male WT mice, suggesting a potential role in mediating resistance to HFD-induced liver injury.

Specifically, *Scd2* was downregulated—a change linked to reduced triglyceride synthesis and adiposity [44, 59]. *Vnn1*, involved in hepatic gluconeogenesis and lipolysis in white adipose tissue, was upregulated; however, its role in liver steatosis remains ambiguous, with some studies associating its presence with steatosis and others with protection under starvation conditions [60, 61]. *Fabp5*, expressed in macrophages, and its deletion are associated with an increase in inflammatory cytokines [62]. It was downregulated in Ames Dwarf females, Ames Dwarf males, and WT females on HFD—suggesting an anti-inflammatory phenotype in these groups. The only gene that showed an exacerbating association was *Lepr*, the leptin receptor gene, which was downregulated and is associated with the progression of MASLD [63].

The robust hepatic protection observed in female Ames Dwarf mice—despite low estrogen signaling- suggests that intrinsic genetic or metabolic adaptations, possibly linked to disrupted GH signaling, confer resilience to metabolic stress. This is consistent with previous studies suggesting that the GH axis modulates sexually dimorphic downstream pathways [64], with compensatory mechanisms in females that are distinct from those in males, as reflected by the more extensive transcriptomic changes observed in male Ames Dwarf mice under HFD.

Interestingly, common gene analysis revealed that female WT and male Ames Dwarf mice on HFD clustered together, suggesting a shared transcriptional response to HFD, which could be due to the lower levels of testosterone in them. This clustering may reflect conserved mechanisms of metabolic compensation, despite differences in the hormonal milieu. Heatmap analysis of commonly regulated genes identified *Clstn3*, *Gpc1*, and *Plin4*—genes involved in lipid droplet formation [65] and membrane dynamics, as potential mediators of these similarities.

Pathway enrichment analysis of uniquely expressed genes highlighted activation of the PPAR and AMPK signaling pathways in male Ames Dwarf mice. These pathways are critical regulators of lipid oxidation, insulin sensitivity, and mitochondrial biogenesis [66, 67]. Downregulation of lipogenic genes [34, 43, 68, 69](*Scd1, Scd2, Acaca*) and signaling components [70, 71] (*Irs2, Ugt1a5*) further supports a metabolic state resistant to steatosis. Cdkn1 and Elovl3 were among the top 10 differentially regulated genes, which are involved in cell cycle repair, and were upregulated in a synchronous manner. Cdkn1a is involved in cell cycle inhibition and cellular senescence, and the literature indicates its association with the progression of MASLD [72, 73]. Elovl3 is an enzyme involved in the elongation of very long-chain fatty acids. In embryonic development, its increased expression has been shown to suppress cell proliferation [74]. The dual upregulation of CdKn1a and Elovl3 under a high-fat diet could be a compensatory response to oxidative and metabolic stress, by limiting hepatocyte proliferation to further dampen the cellular damage. This molecular profile contrasts sharply with WT males, where genes linked to stress and proliferation [33, 35–38, 75] (*Myc, Pde4d, Uap1l1, Mthfd1l, Smpd3*) were upregulated, suggesting a pathophysiological trajectory towards steatosis and hepatocellular carcinoma.

Cytochrome P450 (CYP450) enzymes play a central role in the metabolism of steroids, fatty acids, and xenobiotics, and their hepatic expression is known to be highly sexually dimorphic [76]. This dimorphism is largely regulated by growth hormone, which is secreted in a sex-specific manner—pulsatile in males and continuous in females—resulting in male-biased expression of isoforms such as *Cyp2c11* and *Cyp2a2*, and female-biased expression of *Cyp2c12* [64]. However, in GH-deficient Ames Dwarf mice, this sexual dimorphism in CYP expression is largely abolished due to the absence of GH signaling.

Our transcriptomic analysis reveals distinct, group-specific regulation of CYP450 genes in response to a HFD, particularly within the *Cyp4a* family. In male Ames Dwarf mice on high fat, *Cyp4a12a* and *Cyp4a12b* were significantly upregulated, suggesting a shift toward pathways that favor fatty acid β-oxidation and reduce fibrotic susceptibility; these isoforms are also involved in retinol metabolism and may enhance antifibrotic effects by promoting natural killer (NK) cell-mediated clearance of hepatic stellate cells [42, 77]. In contrast, wildtype male mice on HFD showed upregulation of *Cyp4a14* and *CD36*—a gene pair associated with increased hepatic triglyceride accumulation and inflammation [78]. Notably, *Cyp4a14* was also upregulated in HFD-fed wildtype females, but without a corresponding increase in *CD36*. As mentioned above, CD36 regulation was linked with PPARγ and Pde4d, which were only upregulated in the male WT mice, suggesting that *CD36*, along with its regulators PPARγ and Pde4d, may be a critical determinant of lipid accumulation and inflammatory responses. Additionally, the PPAR signaling pathway was differentially expressed across all groups in response to HFD, but the downstream targets varied. In male Ames Dwarf mice, downregulation of *Scd1* was observed, a gene whose suppression is known to activate AMPK signaling. This activation leads to decreased expression of *Acacb* (acetyl-CoA carboxylase beta), ultimately enhancing fatty acid oxidation [68]. Taken together, our findings suggest that the absence of GH-driven sexual dimorphism in Ames Dwarf mice results in a reprogrammed hepatic CYP450 expression profile that promotes lipid oxidation and attenuates inflammation and fibrosis. This reprogramming may underlie the metabolic resilience of Ames Dwarf mice under HFD stress, emphasizing the intricate link between GH signaling, CYP450 isoforms, and liver health.

Transcriptomic analysis of high-fat diet–fed wild-type and Ames Dwarf mice revealed differential regulation of genes involved in metabolic pathways. Among these, *Fmo3* expression was elevated in both male and female Ames Dwarf mice compared with their wild-type counterparts under high fat diet conditions. *Fmo3* encodes a flavin-containing monooxygenase that catalyzes the oxygenation of sulfur- and nitrogen-containing xenobiotics. Ames Dwarf mice inherently exhibit higher baseline expression of *Fmo3*, which has been linked to their extended lifespan [79]. However, increased *Fmo3* activity has also been associated with the progression of MASLD through reduced lipolysis and enhanced lipogenesis [80]. In our study, *Fmo3* expression did not differ significantly between standard diet– and high fat diet–fed Ames Dwarf mice, suggesting that these mice maintain a constitutively elevated metabolic threshold for this enzyme compared with wild-type mice. In contrast, female wild-type mice displayed a downregulation of *Fmo3* in response to high-fat feeding, which may represent a protective adaptive response to mitigate lipid overload^81^. A major limitation of our study is the lack of functional validation for the genes identified as contributing to the metabolic resilience of Ames Dwarf mice against high fat diet–induced liver injury. While our findings highlight key pathways linked to lipid metabolism and reduced fibrosis, direct evidence linking these genes to growth hormone deficiency is currently lacking. Additionally, our study was limited to young adult mice, leaving open the question of whether observed metabolic protection persists with age. Given that MASLD is strongly age-associated and often worsens with declining insulin sensitivity, future studies should examine how aging influences hepatic gene expression and metabolic outcomes in Ames Dwarf mice. Understanding whether their unique longevity-associated adaptations confer sustained protection against MASLD across the lifespan will provide critical insight into the interplay between aging, GH signaling, and liver health.

## Conclusion

This study provides comprehensive evidence that long-lived GH-deficient Ames Dwarf mice exhibit robust protection against HFD–induced liver pathology. Through integrated histological, inflammatory, and transcriptomic analyses, we show that this metabolic resilience is characterized by suppression of lipogenic and pro-inflammatory signaling, along with modulation of protective pathways such as PPAR and AMPK. These adaptations highlight a distinct set of genes that may serve as potential therapeutic targets for MASLD. Given that MASLD is strongly age-associated and that Ames Dwarf mice maintain insulin sensitivity and metabolic health into old age, our findings offer a unique lens through which to explore aging-related mechanisms of liver protection. Notably, the modulation of PPAR signaling in these mice aligns with the mode of action of current PPAR agonists used clinically to treat MASLD, suggesting that naturally enhanced PPAR activity—modulated by GH deficiency—may underline the sustained hepatic protection observed in these long-lived mice (Table 1).

**Table 1:** Literature association with MASLD pathophysiology for differentially expressed genes across the eight groups used for RNAseq analysis and possible mechanism of action and KEGG pathway mapped using ShinyGo analysis. (NS = Transcripts were detected in the group, but didn’t reach the filter criteria for differentially expressed genes)

	Male	Female	Male	Female	Male	Female	Male	Female		
Gene	DF STD vs WT STD	DF STD vs WT STD	DF HFD vs DF STD	DF HFD vs DF STD	WT HFD vs WT STD	WT HFD vs WT STD	DF HFD vs WT HFD	DF HFD vs WT HFD	Mechanism	Kegg pathway
Abcc3	NS	NS	NS	NS	1.51267744334598 (Exacerbating)	NS	NS	NS	The transporter protein plays a role in bile acid transport and detoxification. Upregulated with NASH. [82]	Bile secretion, ABC transporters
Abcc4	3.1455237878774	NS	NS	NS	NS	NS	1.80537696035769	NS	Inhibition of hepatocyte Abcc4 increases hepatic low-density lipoprotein receptor abundance and enhances low-density lipoprotein clearance. Inhibitor of Abcc4 in high-fat diet mice model improved plasma low-density lipoprotein cholesterol. [83]	Bile secretion, ABC transporters
Acot1	1.6457126433534 (Protective)	NS	1.70506130697338 (Protective)	NS	NS	NS	2.13153626271504 (Protective)	NS	Converts acyl-CoA to fatty acid and CoA. It regulates PPARα reporter activity and regulates hepatic fatty acid metabolism by balancing oxidative flux capacity [67]	Biosynthesis of unsaturated fatty acid, Fatty acid elongation, Metabolic pathway
Acot2	3.29517979161936	NS	NS	NS	2.7983722994211	1.555462373	1.7991607622574	NS	Localized in mitochondria. Its upregulation can enhance the hepatic oxidation of fatty acids. [84]	Biosynthesis of unsaturated fatty acid, Fatty acid elongation, Metabolic pathway
Acot3	6.20702667884707 (Exacerbating)	NS	NS	NS	2.41986551160055 (Exacerbating)	NS	3.80189529741647 (Exacerbating)	NS	Localized in the peroxisome. Hydrolyze long-chain acyl-CoA to free fatty acid and CoA. The literature shows an association with hepatic steatosis with upregulation in Acot3 during chronic exposure to nano and microplastics. [40, 49]	Biosynthesis of unsaturated fatty acid, Fatty acid elongation, Metabolic pathway
Acot4	2.86767646600674 (Exacerbating)	NS	NS	NS	NS	NS	2.25528133178177 (Exacerbating)	NS	Acot4 upregulation inhibited AMPK activity by regulating the phosphorylation of AKT. It also impacts insulin sensitivity [85]	Biosynthesis of unsaturated fatty acid, Fatty acid elongation, Metabolic pathway
Acaca	NS	NS	−1.76873865809184 (Protective)	NS	NS	NS	NS	NS	Involved in regulating the rate of fatty acid synthesis. Silencing of this gene showed reduced intracellular lipid accumulation of triglyceride along with a decrease in mitochondrial dysfunction and oxidative stress. [34]	AMPK Signaling, Fatty acid metabolism, Pyruvate metabolism, Propanoate metabolism, Alcoholic liver disease, Metabolic pathways
Acacb	NS	NS	−2.18838096322461 (Protective)	NS	NS	NS	NS	NS	Inhibition of Acc enzyme activity shown to reduced hepatic malonyl-CoA level, enhanced hepatic ketogenesis and reduction in hepatic de novo lipogenesis. [86]	AMPK Signaling, Metabolic pathways
Acss2	NS	NS	−2.04305590173973 (Protective)	NS	NS	−1.639222675 (Protective)	NS	NS	Plays a role in generating acetyl-CoA, which is involved in fatty acid oxidation in mitochondria or lipid synthesis via de novo lipogenesis [87]	Pyruvate metabolism, Propanoate metabolism, Carbon metabolism, metabolic pathways
Acmsd	NS	NS	−1.85961968549879	NS	NS	NS	NS	2.65671520343444	Acmsd inhibition increased NAD + level by accumulating 2-Amino-3-carboxymuconate-6-semialedyhde, which further leads to increase NAD +, which improves mitochondrial function and can reduce hepatic steatosis. [88]	Tryptophan metabolism, Metabolic pathways
Aldh1a1	NS	NS	NS	NS	NS	2.26350805 (Protective)	NS	−1.5336631063176	Plays role in acetaldehyde oxidation and metabolizes retinal to retinoic acid. Low protein level is associated with NASH. [89]	Retinol metabolism, Metabolic pathways
Aldh3a2	NS	NS	NS	NS	NS	2.02983198348622	NS	NS	Convert fatty aldehydes to fatty acid and detoxify oxidized lipid species. [55, 56, 89]	Fatty acid degradation, Pyruvate metabolism, Tryptophan metabolism, Glycerolipid metabolism, Alcoholic liver disease
Anxa2	NS	NS	NS	NS	2.14252055020253	1.9265009	NS	−2.04134920498511	Associated with MAFLD along with lipid accumulation and fibrosis. Along with the marker of MASH progression. [90, 91]	
Anxa5	NS	NS	NS	NS	2.37474791242596	NS	NS	−1.88875705758513	Protective by promoting the switch of hepatic macrophages from pro-inflammatory M1 to anti-inflammatory M2 polarization and reducing the MASH progression. [90]	
Apoa4	NS	−2.867881901	NS	NS	NS	NS	−3.09103426334203	−3.80118444231632	Expression of this protein is increased with high-fat intake, and its expression is tightly regulated by triglyceride. [92]	Fat digestion and absorption, Cholesterol metabolism
CD36	NS	NS	NS	NS	2.10952766058021 (Exacerbating)	NS	NS	NS	Involved in transport of long chain fatty acid from blood to hepatocytes and regulating lipid metabolism via PPARγ and AMPK pathway. Upregulation can lead to increase lipid deposition and fibrosis. [36, 45, 93]	PPAR Signaling, AMPK signaling pathway, Fat digestion and absorption, Cholesterol metabolism
Chrna2	NS	NS	1.71172128515804 (Protective)	2.224885756 (Protective)	NS	2.224720736	NS	NS	Chrna2 knockout mice tend to have higher susceptibility to diet-induced MASH. Its deficiency leads to increased SREBP1 maturation and phosphorylation of C-Jun NH2-terminal kinase and NFKB. [94]	
Chrna4	NS	−3.539537391	−4.2255237468232 (Protective)	NS	−4.2531638717192 (Protective)	NS	NS	−3.64646282950839	An increased level is observed in mice and patients with MASH. Chrna4 induces calcium influx and activation of inflammatory signaling. [95]	Chemical carcinogenesis-receptor activation
Clstn3	NS	NS	1.97541811974548	NS	2.26212325549326	2.717666642	NS	−2.93164172639521	Clstn3 overexpression modulates lipid metabolism and gluconeogenesis via increasing the FXR signal pathway. [65]	
Cyp1a2	NS	NS	NS	NS	−1.77361202228401	NS	NS	1.60372961647678	Responsible for drug metabolism and xenobiotic detoxification and can contribute to oxidative stress. Studies have shown downregulation of Cyp1a2 in NAFLD. [54, 96]	Steroid hormone biosynthesis, Retinol metabolism, Tryptophan metabolism, Drug metabolism-cytochrome P450, Chemical carcinogenesis-receptor activation, Metabolic pathways
Cyp17a1	4.55169534344898	NS	−2.18110392313388	NS	NS	NS	2.41277535734686	NS	Could be linked to reduce clearance of drug. [97, 98]	Steroid hormone biosynthesis, Metabolic pathways
Cyp2b9	6.04051572609339	NS	NS	NS	4.96074077738719	NS	NS	NS	Increases in NASH groups could be contributing to oxidative stress by generating superoxide radicals. [99]	Arachidonic acid metabolism, Steroid hormone biosynthesis, Retinol metabolism, Chemical carcinogenesis-receptor activation, Metabolic pathways
Cyp2b13	9.6955600373301	3.897025801	NS	NS	4.84707251898828	3.800996124	5.5987560254408	NS	Cyp2b null male mice showed mild increase in steatosis and one of the cyp2b increase was cyp2b13. [100]	Arachidonic acid metabolism, Steroid hormone biosynthesis, Retinol metabolism, Chemical carcinogenesis-receptor activation, Metabolic pathways
Cyp4a12a	−6.1876786211163	NS	4.84727510415 (Protective)	NS	NS	NS	NS	NS	Usually, females are more protective. Regulated by PPARα by beta oxidation of long chain fatty acid. [77]	PPAR Signaling, Retinol metabolism, Arachidonic acid metabolism, Fatty acid degradation, Inflammatory mediator reg. of TRP channels, Metabolic pathways
Cyp4a12b	−7.01086870251868	NS	4.5892834074336 (Protective)	NS	NS	NS	NS	NS	Reduce the development of fibrosis via the retinoic pathway. [99]	PPAR Signaling, Retinol metabolism, Arachidonic acid metabolism, Fatty acid degradation, Inflammatory mediator reg. of TRP channels, Metabolic pathways
Cyp4a14	7.01763055473124	3.47503238	NS	NS	4.63447851535851 (Exacerbating)	5.217042843 (Exacerbating)	3.47903581049873 Catalyzes omega-hydroxylation of medium-chain fatty acid and arachidonic acid. Upregulation leads to NAFLD with increased CD36 expression	NS	Catalyzes omega-hydroxylation of medium-chain fatty acid and arachidonic acid. Upregulation leads to NAFLD with increased CD36 expression. [78]	PPAR Signaling, Retinol metabolism, Arachidonic acid metabolism, Fatty acid degradation, Inflammatory mediator reg. of TRP channels, Metabolic pathways
Cyp51	NS	NS	−1.59820801839936 (Exacerbating)	NS	NS	NS	NS	NS	Involved in cholesterol synthesis. Hepatocyte knock out of Cyp51 mice develops hepatomegaly, fibrosis, and inflammation, but without steatosis. [98, 101]	Steroid biosynthesis, Metabolic pathways
Cyp7b1	−4.80928120419217	NS	NS	NS	NS	NS	−3.22916853336535	NS	Decreased expression is seen in the mouse and human MASLD conditions. Its transgenic expression is seen and prevents liver toxicity by suppressing the accumulation of bioactive oxysterols such as (25R)26-hydroxycholesterol (26HC) and 25-hydroxycholesterol (25HC). [102]	Steroid hormone biosynthesis
Cdkn1a	−2.19490624324737	−3.592358899	2.82126776977454 (Exacerbating)	NS	1.70789066019402 (Exacerbating)	NS	NS	−2.82002148384482	Cell cycle inhibitors may be regulated in response to DNA and oxidative stress [73]	Pathways in cancer
Ces1d	1.96346339008202	NS	NS	NS	NS	NS	2.21514838377359 (Should be exacerbating but comparing to DF mice on HFD vs STD ACC enzyme (Acaca, Acacb) were downregulated, which is protective	NS	Ces1d deficiency is associated with activation of AMPK and inhibitory phosphorylation of acetyl-CoA carboxylase. [103]	
Ces1g	NS	NS	NS	NS	NS	NS	1.89226116626356	NS	Ces1g is an important regulator of lipid metabolism, and its deficiency can lead to impaired insulin sensitivity. [104]	
Ces3b	−3.65166540720098	−3.309425855	NS	NS	NS	NS	−3.71242000334606	−1.80987431896617	In MASH patients, hepatic Ces3 mRNA and protein were significantly reduced. But overexpression of hepatic Ces3 aggravated the western diet induced MASH, suggesting its role in progression of MASH and liver reduces its expression in the liver to prevent lipid accumulation in MASH. [105]	
Cgref1	NS	NS	NS	NS	3.01462350161848	NS	NS	NS	A chronic high-fat diet can induce expression could induce insulin resistance in the white adipose tissue which can elevate blood glucose, free fatty acid level and increase de novo lipogenesis and promote hepatic lipid accumulation. [106]	
Cidea	−4.25631514137355	NS	4.77033776774285 (Contribute to obesity and correlates with liver steatosis progression)	7.44872310156027 Contribute to obesity and correlates with liver steatosis progression)	NS	NS	−6.93470047519097	NS	Regulate lipid droplet growth and VLDL production. Tends to increase with obesity as well as decrease with steatosis severity [41]	AMPK Signaling
Cidec	NS	NS	NS	NS	5.12775993556575	NS	NS	NS	Upregulation is associated with the accumulation and increase in size of lipid droplets. Its expression is increased in HFD treatment via PPARγ. [41]	
Col12a1	NS	NS	NS	NS	1.64902849053244	NS	NS	NS	Literature has shown upregulation in intrahepatic cholangiocarcinoma and colorectal liver metastasis. Due to its role in collagen I fibrils and surrounding matrix interaction, it plays significant role in maintaining structural integrity of liver and might be related to hepatocyte damage in the liver. [107]	
EGFR	−5.28421972373947	−1.621048369	NS	NS	−2.19177438076456	NS	−3.10965003310955	NS	Regulates cell proliferation, regeneration, oxidative stress, and apoptosis. Might reduce hepatocyte proliferation and prevent hepatic stellate cells activation. [108, 109]	Pathways in cancer, Ras signaling pathway, MAPK signaling pathway, PI3K-Akt signaling pathway
Elovl3	−8.37499974364528	−2.78972765	4.99433502301073 (No impact)	NS	NS	3.25557837 (No impact)	−2.06940972992783	−4.69219938665979	Catalyze the synthesis of C20-24 fatty acid, and its deficiency is associated with an anti-obesity effect in mice. [56, 74, 110]	Biosynthesis of unsaturated fatty acids, Fatty acid elongation, Fatty acid metabolism, Metabolic pathways
Elovl6	NS	NS	**-**2.20617362072037 (Protective)	NS	NS	−2.266365693 (Protective	NS	NS	Involved in the elongation of C12-16 saturated and monounsaturated fatty acids. It is involved in progression of NASH, and its deletion reduces palmitate-induced activation of the inflammation [111, 112]	Biosynthesis of unsaturated fatty acids, Fatty acid elongation, Fatty acid metabolism, Metabolic pathways
Enpp2	NS	2.436025905	NS	NS	NS	1.709970868	NS	NS	Increase lipid oxidation leads to migration and proliferation of hepatic stellate cells that can lead to liver fibrosis. [110]	Ether lipid metabolism, Metabolic pathways
Etnppl	NS	NS	NS	NS	NS	−1.50707463	NS	1.93255019653824	Enzyme degrades phosphoethanolamine an intermediate in Phosphatidylethanolamine synthesis. Etnppl knockout mice have higher fasting total plasma cholesterol, triglyceride, and apolipoprotein, but not the hepatic triglyceride secretion. The impact is more metabolic change. [113]	Metabolic pathways
Fabp5	NS	−4.2025952	−3.55867569275384	−2.492001236	NS	−4.836380491	NS	−1.85821594469879	Regulators of macrophage phenotype in acute injury and Fabp5 knockout mice showed higher mRNA levels of anti-inflammatory cytokines. [62, 114]	PPAR signaling pathway
Fdps	NS	NS	−1.73567967850915 (Protection)	NS	NS	NS	NS	NS	FDPS generates farnesyl pyrophosphate (FPP), which acts as an agonist of aryl hydrocarbon receptor and upregulates the expression of CD36, leading to the development of NASH. Inhibition of fdps can reduce fpp load and impact the progression of NASH. [45]	Metabolic pathways
Fgf21	−2.23100020421243	−3.420078569	NS	NS	NS	NS	−2.2001751051833	−2.1467152791962	Starvation hormone and associated with lipolysis. [115–117]	Pathways in cancer, Ras signaling pathway, MAPK signaling pathway, PI3K-Akt signaling pathway
Fmo2	3.47840473804965	NS	NS	NS	NS	NS	2.00546643583009	1.52516419783192	Fmo2 disrupts lipogenesis by binding to SREBP1 and inhibiting its translocation from the endoplasmic reticulum to the Golgi apparatus and further signaling. [40, 118]	Taurine and hypotaurine metabolism, Drug metabolism-cytochrome P450, Metabolic pathways
Fmo3	10.1531507132604	NS	NS	NS	NS	−5.978437364 (Could be protective)	9.97832528710078	6.65198512302018	Downregulation is protective by against obesity by beiging of white adipose tissue regulating the hepatic lipid level via trimethylamine-N-oxide [81, 119]	Taurine and hypotaurine metabolism, Drug metabolism-cytochrome P450, Metabolic pathways
Gadd45a	2.42946690757532	NS	NS	NS	1.70192758505804 (Protective)	NS	NS	NS	Activate under stress conditions. Gadd45a may exert protection against hepatic fibrosis by inducing CCl4 and inhibiting TGFβ/Smad signaling. [120–122]	Pathways in cancer, p53 signaling pathway, FoxO signaling pathway, MAPK signaling pathway
Gpc1	NS	−3.68403701114694	4.70533695606193 (Exacerbating)	NS	1.94572645569576 (Exacerbating)	4.000512559 (Exacerbating)	NS	−2.5996736677505	Highly expressed in hepatocellular carcinoma. [123]	Proteoglycans in cancer
G6pdx	NS	−2.682415913	−1.78054539074387 (Compensating, metabolic impact)	NS	NS	−2.713951294 (Compensating, metabolic impact)	NS	NS	Produce NADPH, which is involved in the biosynthesis of fatty acids and cholesterol. Deficiency of G6pd is linked decrease weight gain and hyperinsulinemia but elevates serum fatty acid, without affecting glucose tolerance. [124]	Central carbon metabolism in cancer, Metabolic pathways, Carbon metabolism
Got1	NS	NS	−2.11331335267723 (Protective)	NS	NS	NS	NS	NS	Upregulated in many cancers and can generate an excess of NADPH and impact the redox balance. GOT1 inhibitors can inhibit cancer cell proliferation by impacting ROS. [125]	Arginine and proline metabolism, Metabolic pathways, Carbon metabolism Steroid hormone biosynthesis
Hmgcr	NS	NS	−2.13237342213698 (Protective)	NS	−1.90317131814127 (Protective)	NS	NS	NS	Increased expression is associated with NAFLD via increasing cholesterol synthesis [126]	AMPK signaling pathway, Bile secretion, Metabolic pathways,Bile secretion
Hsd3b5	−10.817481912916	−7.375975316	NS	NS	−5.02131147504607 (Exacerbating)	−6.007543453 (Exacerbating)	NS	NS	Enzyme involved in the conversion of △5-steroids to △4-ketosteroids and regulates lipid homeostasis, and is associated with steatosis. [127]	Steroid hormone biosynthesis, Metabolic pathways,
Igf1	−3.25915606367843	−3.300035459	NS	NS	NS	NS	−2.03579354697174	NS	Igf-1 mRNA expression was significantly lower in patients with hepatic steatosis and NAFLD activity score. [128]	Pathways in cancer, AMPK signaling pathway, p53 signaling pathway, EGFR tyrosine kinase inhibitor resistance, Endocrine resistance, FoxO signaling pathway, HIF-1 signaling pathway
Igfbp2	NS	NS	−3.30566363641688 (Exacerbating)	NS	−1.68464546276153 (Exacerbating)	NS	NS	2.59805923580021	Studies have shown a decrease in mRNA expression associated with MASLD. With one of the study showed its overexpression can decrease oleic acid induced triglyceride accumulation in HepG2 cells [91]	
Impa2	NS	NS	NS	NS	1.76274566735041 (Could be protective)	NS	NS	NS	Downregulation is associated with colorectal cancer and liver metastasis. [129]	Metabolic pathways
Insig1	NS	NS	NS	NS	−1.5751206091603 (Protective)	−1.624768432 (Protective)	NS	NS	Loss of Insig1 promotes lipid remodeling and prevents hepatic lipotoxicity [130]	
Irf6	−1.58399556119191	−1.915911913	NS	NS	NS	NS	−1.8847383371309 (Exacerbating)	NS	Irf6 binds to the promoter of PPARγ and inhibits its transcription thus regulating lipogenesis and lipid uptake. It was downregulated in high-fat diet induced fatty liver via promoter hypermethylation. [131]	
Lepr	3.28148198782389	NS	−2.88886117262143 (Exacerbating)	−1.625403022 (Exacerbating)	NS	−3.206786187 (Exacerbating)	NS	2.27263325340587	In serum, NAFLD patients tend to have lower levels [63]	AMPK Signaling, JAK-STAT signaling pathway
Ly6d	NS	NS	2.95217679812422 (Exacerbating)	NS	5.503206336434 (Exacerbating)	NS	NS	−4.66862975965695	Expression is significantly higher in HFD mice. It regulates hepatic steatosis by phosphorylation of ATP citrate lyase. [127, 132]	
Mthfd1l	NS	2.152369698	NS	NS	1.66737399564779 (Exacerbating)	NS	NS	NS	Upregulation is associated with hepatocellular carcinoma. [37]	Metabolic pathways
Mogat1	NS	NS	NS	NS	NS	4.793334214 (Metabolic impact, could impact the weight gain)	NS	−4.32846344348697	Act as ab enzyme to convert monoacylglycerol to diacylglycerol. Its inhibition improves glucose tolerance, weight gain and insulin signaling but didn’t impact the liver inflammation. [133]	Glycerolipid metabolism, Metabolic pathways
Myc	NS	NS	NS	NS	2.41424617421662 (Exacerbating)	NS	−3.26759219503819	NS	Promote hepatic cell proliferation. Upregulation of hepatic c-myc is seen in liver fibrosis in man and mice. [75, 134, 135]	Pathways in cancer, ErbB signaling pathway, Central carbon metabolism in cancer, JAK-STAT signaling pathway, MAPK signaling pathway, PI3K-Akt signaling pathway
Nrg1	NS	NS	NS	NS	1.9276681247253 (Protective)	NS	−2.22729181617843 (Exacerbating)	NS	Ligand for ErbB3 signaling. It can alleviate steatosis via inhibiting IL-6 and upregulating ErbB3 phosphorylation and increasing the expression of PI3K and phosphorylated AKT. [136]	Erbb3 signaling, EGFR tyrosine kinase inhibitor resistance
Nrg4	2.57940759498747	NS	NS	NS	NS	NS	1.76577362932334 (Nrg4 upregulation could be compensating for elevated NRg1 downregulation)	NS	Ligand for ErbB4 receptor tyrosine kinase, which will attenuate hepatic lipogenesis. This may suggest compensating Nrg1 regulation. [137]	
Osbpl3	3.07922282640572	3.574916231	NS	NS	2.94956709527368 (Exacerbating)	3.285120992 (Exacerbating)	NS	NS	High expression is seen in advanced human NAFLD patients. Its expression is regulated via PPARγ and enhanced its transcription by binding to the PPARγ responsive element. [138]	
Pde4d	NS	NS	NS	NS	1.6004589063122 (Exacerbating, directly linking to CD36 upregulation)	NS	NS	NS	Hydrolyze cAMP to AMP, leads to reduced cAMP and upregulate CD36 which leads to increased lipid deposition. [36]	Metabolic pathways, Parathyroid hormone synthesis secretion and action
PPARγ	NS	NS	NS	NS	1.75145678264338 (Exacerbating)	NS	NS	NS	Nuclear receptor and transcription factor that regulates lipid and insulin signaling. Increased fatty acid uptake, binding, and transport could lead to an increase steatosis. [53, 66, 139]	PPAR Signaling
Plaat3	NS	1.73328455982183	NS	NS	NS	NS	1.83790788366483 (Protective)	NS	Highly expressed in adipose tissue and it’s a ligand of PPARγ regulator. Plaat3 deficient mice have lower accumulation of fat in white adipose tissue but ectopic accumulation of hepatic fat and increased insulin resistance. [140, 141]	Arachidonic acid metabolism, Ether lipid metabolism, Metabolic pathways, Ras signaling pathway
Pnpla3	NS	−4.074250748	NS	NS	NS	−4.229602099 (Protective)	NS	NS	Involved in triglyceride remodeling and lipid turnover. Mutation 148 M is well studied. Carbohydrate intake should increase Pnpla3, along with increased fatty acid synthesis, which suggests a compensation. [142–144]	Glycerolipid metabolism, Metabolic pathways
Ppp1r3b	NS	NS	NS	NS	NS	NS	1.67747892701872 (Protective)	NS	It is a crucial regulator of hepatic metabolism and is associated with liver glycogen synthesis and maintaining glucose level. Its overexpression is associated with increased glycogen storage and deletion in higher lipid accumulation, [145]	
Ptges	−6.56870481695951 (Protective)	NS	NS	NS	NS	NS	−5.79377894900313 (Protective)	NS	Hepatic mRNA expression of Ptges is higher during hepatic ischemia perfusion liver injury. Ptges is a modulator of inflammation and mice with Pteges deletion showed faster injury repair and enhanced regenerative marker like HGF, EGF, and VEGF. [146]	Arachidonic acid metabolism, Metabolic pathways
Scd1	NS	NS	−1.63861705025817 (Protective)	NS	NS	NS	NS	NS	Enzymes involved in synthesis of monounsaturated fatty acid from saturated fatty acid and its deficiency increase the saturation of liver lipid species and could lead to hepatic fibrosis in high carb diet and low fat diet. On the contrary, Scd1 inhibitor in high-fat diet induced NAFLD, improved the glucose tolerance and reduced lipid accumulation. The inhibitor acti via suppressing hepatic lipogenesis and adaptogenic differentiation via SCD1-ATF3 signaling. [43, 68, 69]	Biosynthesis of unsaturated fatty acids, Fatty acid metabolism, PPAR signaling pathway, AMPK signaling pathway, Alcoholic liver disease, Metabolic pathways
Scd2	4.53746994027768 (Could be contributing to Ames Dwarf mice higher adiposity compared to wild type)	NS	−4.15208178866147 (Protection)	−3.350222247 (Protection)	NS	NS	NS	NS	Like Scd1, it is also involved in the synthesis of monounsaturated fatty acid. Contrary to Scd1, knockout mice of Scd2 show protection against diet induced adiposity [44]	Biosynthesis of unsaturated fatty acids, Fatty acid metabolism, PPAR signaling pathway, AMPK signaling pathway, Alcoholic liver disease, Metabolic pathways
Smpd3	1.66313777282754	NS	NS	NS	1.94415917778845 (Exacerbating)	NS	NS	NS	Involved in caveolae-dependent lipid uptake and extracellular vesicle release. Lipotoxicity can induce SMPD3 via SIRT1 signaling and disrupt sphingomyelin ceramide balance in the membrane and promote steatosis by enhancing caveolae-dependent lipid uptake and extracellular vesicle secretion. [38]	Metabolic pathways
Sult1e1	3.43104002658794	1.51932015	NS	NS	NS	NS	3.20796566396599	NS	Associated with no change in steatosis, diabetic cirrhosis, and a decrease in alcohol cirrhosis. [147]	Steroid hormone biosynthesis, Metabolic pathways
Sult2a1	11.750191163089	NS	−2.79974696387289 (Could be exacerbating)	NS	NS	NS	9.63205596553592	NS	Associated with no change in steatosis, diabetic cirrhosis, and a decrease in alcohol cirrhosis. [147]	Bile secretion
Tm7sf2	NS	−1.64603388921919	NS	NS	NS	NS	NS	NS	This gene disruption can affect the cholesterol synthesis pathway via regulating Cyp51. [72]	Steroid biosynthesis, Metabolic pathways
Uap1l1	NS	NS	NS	NS	1.83829802774903 (Exacerbating)	NS	NS	−1.56272270505364	Upregulation is associated with hepatocellular carcinoma and modulates OGlcNAcylation of c-Myc. [35]	Metabolic pathways
Vldlr	5.68026955995263	2.359927271	NS	NS	NS	2.42424690075115	2.414372029558	1.58337588858234	The regulation of Vldlr depends upon the type of PPAR signaling. Literature has shown that PPARα activation upregulates Vldlr and could reduce triglyceride. Studies have also shown that PPARβ/δ deficiency increased hepatic Vldlr level and, in human hepatic steatosis, increased the level of Vldlr and reduced expression of PPARβ/δ/δ. [148, 149]	
Vnn1	NS	−2.752359339	NS	2.480843241	NS	3.505124009	−3.77664010662864	NS	Enhances gluconeogenesis and increases reactive oxygen species (ROS) production via the cysteamine pathway. [60, 61]	Pantothenate and CoA biosynthesis, Metabolic pathways

## Supplementary Information

Below is the link to the electronic supplementary material.

ESM 1 (TIF 342 KB)

ESM 1 (PNG 144 KB)

ESM 2 (TIF 867 KB)

ESM 2 (PNG 503 KB)

ESM 3 (XLSX 175 KB)

## Data Availability

The datasets can be accessed on the NCBI under BioProject PRJNA1305481 at the following link: https://www.ncbi.nlm.nih.gov/sra/PRJNA1305481.
